# Remediation of PAH-Contaminated Soil by Combining Surfactant Enhanced Soil Washing and Iron-Activated Persulfate Oxidation Process

**DOI:** 10.3390/ijerph16030441

**Published:** 2019-02-02

**Authors:** Yanhua Qiu, Meilan Xu, Zongquan Sun, Helian Li

**Affiliations:** School of Water Conservancy and Environment, University of Jinan, Jinan 250022, China; Qiu_Yan_Hua@163.com (Y.Q.); milan_lyene@163.com (M.X.); sun_zongquan@163.com (Z.S.)

**Keywords:** soil remediation, PAHs, SDS, SiO2/nZVI, activated persulfate

## Abstract

There is increasing concern regarding soils contaminated with polycyclic aromatic hydrocarbons (PAHs)**.** In the present study, the remediation of soil spiked with PAHs was explored by the combination of soil washing with sodium dodecyl sulfate (SDS) and subsequent oxidation through persulfate (PS) activated by Fe^2+^, nanoscale zero-valent iron (nZVI), and SiO_2_-coated nZVI (SiO_2_/nZVI). Results demonstrated that the removal of phenanthrene (PHE), fluoranthene (FLU), and pyrene (PYR) by SDS is an efficient means for soil decontamination. At SDS concentration of 20 g/L, the removal efficiencies of PHE, PYR, and FLU were 37%, 40%, and 44%, respectively. For the degradation of PAHs and SDS in the soil washing effluents, the efficiencies of PS activated with SiO_2_/nZVI were not significantly different from those of PS activated with nZVI and Fe^2+^ (*p* > 0.05). In practice, SiO_2_/nZVI is more preferable due to the improved antioxidation and dispersibility. At the dosage of 2 g/L (in the amount of iron) of SiO_2_/nZVI, the removal efficiencies of PHE, FLU, PYR, and SDS within 30 min of treatment were 75%, 85%, 87%, and 34%, respectively. The degradation of SDS was much lower than those of PAHs, which facilitated the recycle of SDS. Our findings suggest that PS activated with SiO_2_/nZVI is a promising method for the treatment of soil washing effluents containing SDS and PAHs.

## 1. Introduction

Polycyclic aromatic hydrocarbons (PAHs) are among the most widespread persistent organic compounds consisting of two or more fused benzene rings. The dominant sources of PAHs in the environment result from the incomplete combustion of organic matter from human activities and natural sources [1]. PAHs are carcinogenic, mutagenic, toxic for reproduction, and can be accumulated by human and other organisms [2,3]. Due to the characteristics of volatile, low solubility, fat solubility, and high octanol-water partition coefficient, PAHs have a tendency to sorb preferably to soil organic matters. Regarding PAHs removal from contaminated soil or groundwater, there is a wide variety of different techniques that can be applied, such as soil washing with surfactants, chemical oxidation [4,5], and biological and thermal treatments [6]. However, the hydrophobic behavior of PAHs can make their remediation using chemical or biological techniques less suitable. The removal of PAHs from soil by soil washing is a preferable method because it is a simple process with low cost and mild operation conditions. Surfactants are usually used in soil washing because they can enhance the solubility of PAHs, decrease the surface tension, and promote the efficiency of soil washing [7]. Among the surfactants, sodium dodecyl sulfate (SDS) is widely used in soil washing of organic pollutants. It consists of a linear hydrocarbon tail attached to a sulfate group that can be easily released into the solution. Besides, ${\mathrm{SO}}_{4}^{2-}$ released by SDS is readily oxidized to $S_{2}O_{8}^{2-}$ which in turn can be transformed into ${\mathrm{SO}}_{4}^{∙-}$ by activation (Equation (1)) [8,9].

(1) $${\mathrm{SO}}_{4}^{2-}+{\mathrm{SO}}_{4}^{2-}\to S_{2}O_{8}^{2-}$$

However, soil-washing effluents contain a complex mixture of dissolved PAHs and surfactants, which can bring about secondary pollution to the environment if discharged without proper treatment. The methods for the treatment of soil washing wastewater include advanced oxidation process [10], bio-treatment processes [11], electrochemical oxidation [12], photocatalytic processes, and the combination of some of the techniques [8,13]. 

Compared with other treatment processes, advanced oxidation processes that are based on the production of very reactive radicals (OH∙ or ${\mathrm{SO}}_{4}^{∙-}$) are one of the highly effective techniques for treatment of soil washing effluents. For decades, sulfate radicals (${\mathrm{SO}}_{4}^{∙-}$) have been employed to degrade the pollutants in soil and wastewater because of their high redox potential (E^0^ = 2.60 V), which is similar to that of hydroxyl radicals (E^0^ = 2.80 V) [14]. In general, ${\mathrm{SO}}_{4}^{∙-}$ are generated via the activation of persulfate (PS). A variety of activation methods have been researched, including heat, UV-light (Equation (2)), transition metal (Equation (3)), and ultrasound [15].

(2) $$S_{2}O_{8}^{2-}+\mathrm{hv}/\mathrm{heat}\to 2{\mathrm{SO}}_{4}^{∙-}$$

(3) $$S_{2}O_{8}^{2-}{+M}^{n+}\to {\mathrm{SO}}_{4}^{∙-}{+\mathrm{SO}}_{4}^{2-}{+M}^{(n+1)+}$$

Ferrous, as a transition metal, has better advantages to active persulfate (PS) because it could work at room temperature and under atmospheric pressure. However, there are some limitations, such as the rapid conversion of ferrous (Fe^2+^) into ferric (Fe^3+^) and the scavenging of sulfate radicals by Fe^2+^, which are described in Equations (4) and (5) [16]. The use of zero-valent iron or nanoscale zero-valent iron (nZVI) has been considered as the source of Fe^2+^ (Equation (6)) to avoid these problems and to improve the ability to activate PS [17,18]. However, there are still some drawbacks to employing nZVI resulting from the gradually reduced reactivity through oxidation in the air. To address this problem, many measures including the surface coating and supporting of nZVI have been adopted to improve the stability and dispersal of nZVI. Many materials have been used to coat nZVI, such as polyacrylic acid [19], biodegradable polymers including guar gum, potato starch, alginic acid, carboxymethyl cellulose [20], and silica [21]. Among these materials, silica (SiO_2_) has better stability, core-shell, and environmental friendliness characteristics [22].

(4) $${\mathrm{Fe}}^{2+}{+S}_{2}O_{8}^{2-}\to {\mathrm{SO}}_{4}^{∙-}{+\mathrm{SO}}_{4}^{2-}{+\mathrm{Fe}}^{3+}$$

(5) $${\mathrm{Fe}}^{2+}{+\mathrm{SO}}_{4}^{∙-}\to {\mathrm{SO}}_{4}^{2-}+{\mathrm{Fe}}^{3+}$$

(6) $${\mathrm{Fe}}^{0}{+S}_{2}O_{8}^{2-}\to {\mathrm{Fe}}^{2+}{+2\mathrm{SO}}_{4}^{2-}$$

In this study, nZVI and SiO_2_-coated nZVI (SiO_2_/nZVI) synthesized through a liquid reduction method were employed to active persulfate to produce sulfate radicals for the removal of PAHs and SDS from soil washing effluents. Phenanthrene (PHE), fluoranthene (FLU), and pyrene (PYR) were selected to represent PAHs with different hydrophobicity and molecular size due to the fact that they are frequently detected at high concentrations in soils from industrial contaminated sites. The objectives of this study were to (1) obtain the soil washing efficiencies of PAHs by SDS, (2) assess the removal efficiencies of PAHs and SDS with PS activated by different sources of Fe, including Fe^2+^, nZVI, and SiO_2_/nZVI, and (3) obtain the optimal dosages of SiO_2_/nZVI-activated persulfate that should be used in the treatment of soil washing effluents. We propose this as a feasible method for the treatment of washing effluents containing SDS and PAHs in the remediation of contaminated soil.

## 2. Materials and Methods 

### 2.1. Reagents

Phenanthrene (PHE), fluoranthene (FLU), and pyrene (PYR), used for contaminants and HPLC analysis, were purchased from Aladdin Company (Shang Hai, China) with purities of 99%, 98%, and 98%, respectively. Analytical grade sodium dodecyl sulfate (SDS) was used as the surfactant. Potassium borohydride (KBH_4_), ferrous sulfate heptahydrate (FeSO_4_·7H_2_O), chemical grade polyethylene glycol 4000 (PEG-4000), high-purity nitrogen, and ethanol were used to produce nZVI. Tetraethyl orthosilicate (TEOS) with 98% purity and sodium hydroxide (NaOH) were both employed to coat nZVI. Sodium persulfate (PS) was applied as the oxidant. For the analysis of SDS, the reagents required were sodium thiosulfate, methylene blue, anhydrous sodium sulfate, and chloroform. All the reagents were obtained from Sinopharm Chemical Reagent Co., Ltd (Shang Hai, China) unless otherwise noted.

### 2.2. Soil Spiking and Soil Washing 

The soil used in this work was collected from a suburb of Jinan, China. The main soil properties were previously determined by Li et al. [23] and are given in Table 1. The spiked soil was prepared following the method of Zhou and Zhu [24]. Briefly, a methanol solution with PHE, FLU, and PYR was distributed and mixed manually into the soil with a spatula. Then, the solvent was allowed to evaporate slowly in a fume hood. The final concentration of PHE, FLU, and PYR was 100 mg/kg for each, which represented the PAH concentration level of industrial contaminated sites. 

To obtain the PAH removal efficiencies by SDS in the soil washing process, twenty grams of polluted soil were placed in 1-L Erlenmeyer flasks. Then, 200 mL of SDS solutions at different concentrations was added to each flask. The flasks were put on a reciprocating shaker (200 rpm, 25 ± 1 °C) for 48 h. After that, the suspensions were centrifuged, and a specific volume of the supernatant was collected to determine the PAH concentrations.

### 2.3. Synthesis and Characterization of nZVI and SiO_2_/nZVI

nZVI was synthesized through the liquid reduction method using KBH_4_ to reduce Fe^2+^. A quantity of 2.78 g of FeSO_4_·7H_2_O dissolved in 100 mL ethanol/H_2_O (*v*/*v* = 7:3) solution, and 0.5 g of PEG-4000, as the dispersant, were added into a 500-mL three-neck flask. Iron nanoparticles produced were stirred for 30 min while 30 mL of 1 mol·L^−1^ KBH_4_ was dropped into the three-neck flasks at a rate of 2 drops/sec under nitrogen. The synthetic process of SiO_2_/nZVI was similar to previous work [25], with silicon alkoxide as a silica source and amine as a catalyst. A volume of 20 mL of 5 mol/L NaOH and 5 mL TEOS were added to the iron nanoparticle solution then stirred for 1 h. After the reaction, the products were cleaned by deoxygenated water and ethanol three times and dried through vacuum freeze-drying. 

The surface morphologies of nZVI and SiO_2_/nZVI were analyzed using a Nova 450 scanning electron microscope (SEM, USA) at an acceleration voltage of 10 kV. The crystal structures of nZVI and SiO_2_/nZVI were characterized by X-ray diffractometer (XRD, Ultima IV, Japan) with Cu Kα radiation (λ = 1.5408 nm). 

### 2.4. PAHs Degradation in Soil Washing Effluents 

Batch experiments were conducted with soil washing effluents without pH adjustment by using 100 mL Erlenmeyer flasks as reactors equilibrated on a reciprocating shaker at 25 ± 1 °C. Fifty milliliters of soil washing effluents were treated with 10 mL of PS solution (50 mM) containing FeSO_4_, nZVI, or SiO_2_/nZVI synthesized in the same batch. At regular time intervals, a volume of 2 mL sample was removed and filtered through a 0.45 μm PTFE syringe filter. After filtration, 0.020 mol·L^−1^ NaS_2_O_3_ solution (much more than PS) was added to halt the reaction. All these experiments were performed in duplicate. 

### 2.5. Analytical Method

The concentration of SDS was measured following the methylene blue method with a UV-visible spectrophotometer (Shimadzu, Japan) at a wavelength of 650 nm [26]. The concentrations of PAHs were determined by HPLC (Ultimate-3000, Dionex, Sunnyvale, CA, USA) using an ultraviolet detector at a wavelength of 251 nm. The analytical column was a reversed phase SUPELCOSIL LC-PAH column (150 ×4.6 mm, 5 μm). The mobile phase was 90% methanol and 10% deionized water eluted at a flow rate of 1.0 mL·min^−1^. The degradation efficiency of each PAH and SDS was calculated by Equation (7).

(7) $$\mathrm{Degradation}\;\mathrm{efficiency}\;(\%)=\frac{C_{0}-C_{e}}{C_{0}}\times 100$$

*C_0_* is the initial concentration of PAHs or SDS, and *C_e_* is the concentration of PAHs or SDS at equilibrium.

### 2.6. Statistical Analysis 

Statistical analysis was performed using SPSS 22.0 (SPSS Inc. Chicago, IL, USA) software. One-way ANOVA followed by the least significant difference (LSD) test was used to compare the difference among different treatments. Statistical significance was accepted at *p* < 0.05.

## 3. Results and Discussion

### 3.1. The Characterization of nZVI and SiO_2_/nZVI

**Figure 1:**
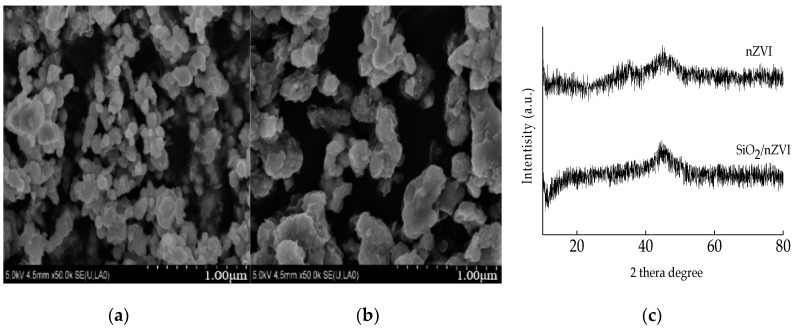
Scanning electron microscope (SEM) images of (**a**) nanoscale zero-valent iron (nZVI), (**b**) SiO_2_-coated nZVI (SiO_2_/nZVI), and (**c**) X-ray diffraction (XRD) patterns of nZVI and SiO_2_/nZVI.

The representative SEM images of nZVI and SiO_2_/nZVI are shown in Figure 1a,b. As can be seen, the shape of synthesized nZVI and SiO_2_/nZVI particles was spherical with the particle size range in the nanoscale. The morphology of nZVI also showed that nZVI formed as chain-like aggregates due to magnetic interaction between small particles. SiO_2_/nZVI has much better dispersion performance than nZVI, suggesting that the coating of SiO_2_ can prevent aggregation by reducing the Van der Waals force and magnetic force between iron nanoparticles. The XRD patterns of the synthesized materials are shown in Figure 1c. From the XRD patterns of nZVI and SiO_2_/nZVI, the diffraction peak at 2 *θ* = 45° confirms the existence of zero-valent iron (α-Fe).

### 3.2. The Washing Efficiencies of PAHs 

**Figure 2:**
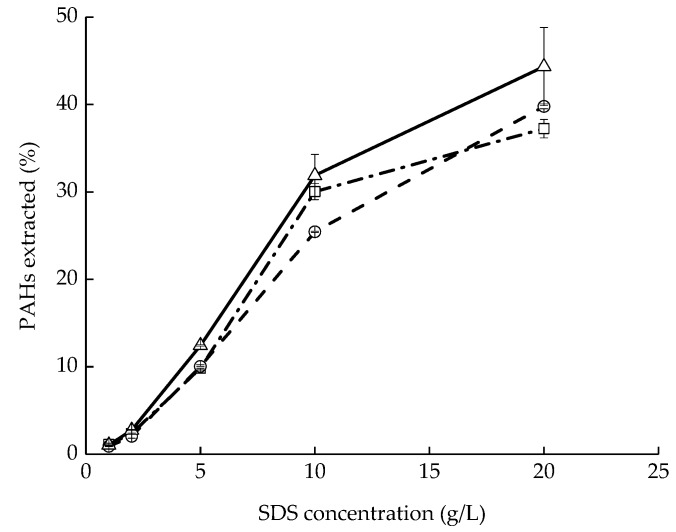
The washing efficiencies of (-·-□-·-) phenanthrene (PHE), (- -○- -) fluoranthene (FLU), and (—△—) pyrene (PYR) at different concentrations of sodium dodecyl sulfate (SDS). Values are presented as means and standard deviation.

In order to assess the removal efficiencies of PAHs from the contaminated soil, soil washing experiments were performed with soils containing PHE, FLU, and PYR at different SDS concentrations. It was not so effective to transfer PAHs from the soil to the liquid phase at low concentrations of SDS (Figure 2). This may be due to the adsorption and precipitation of SDS in the soil-water system, which increased the concentration of SDS required to form micelles that can greatly solubilize PAHs [27]. The removal efficiencies for PHE, FLU, and PYR increased with increasing the concentration of SDS. At a concentration of 20 g/L of SDS, the removal efficiencies of PHE, FLU, and PYR were 37%, 40%, and 44%, respectively. Significant loss of SDS occurred during soil washing process due to the reaction of the cations (Mg^2+^ and Ca^2+^) and the sulfate of SDS [27]. In addition, the soil washing effluent was composed of low concentrations of PAHs and a much higher concentration of SDS. The concentration of PHE, FLU, PYR, and SDS in the washing effluents were 3.8 mg/L, 4.0 mg/L, 4.8 mg/L, and 16 mg/L, respectively. Therefore, the pollution of soil washing effluent was concerned with the high organic load associated with SDS, although the toxicity is mainly owing to the existence of the PAHs rather than SDS. 

In soil washing processes, the removal efficiencies of organic contaminants depend on the characteristics of the specific soil-surfactant system, such as the initial levels of pollutants, the soil properties, and the aging time of pollutants. It was reported that the removal efficiency increased with increasing the initial concentration of organic contaminants due to the increased contact area between the surfactant and the contaminant [28]. Soil properties have an important effect on the removal of PAHs by washing. Zhou and Zhu [24] showed the removal of PAHs was easier in soil with relatively low organic carbon and clay contents. Moreover, longer aging time resulted in decreased removal efficiencies of soil washing.

### 3.3. Effect of the DegradationTime

**Figure 3:**
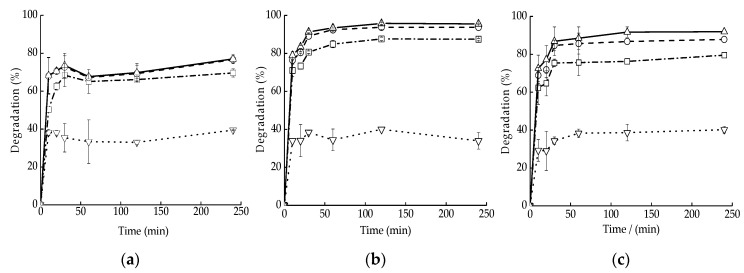
The degradation efficiencies of (-·-□-·-) PHE, (- -○- -) FLU, (—△—) PYR, and (····▽····) SDS by (**a**) Fe^2+^, (**b**) nZVI, and (**c**) SiO_2_/nZVI activated persulfate (PS) with time. Values are presented as means and standard deviation.

Figure 3 describes the degradation efficiencies of PAHs and SDS by iron-activated PS with time. As can be seen, for the PS activated by 2 g/L (in the amount of iron) of SiO_2_/nZVI, nZVI, and Fe^2+^, the degradation rates of PAHs increased rapidly at the beginning of the experiment, with more than 60% removal occurring in the initial 10 min. It indicated that $S_{2}O_{8}^{2-}$ can be activated to generate a large amount of ${\mathrm{SO}}_{4}^{∙-}$ in a short time. After that, the degradation proceeded much slower, reaching equilibrium at 30 min. The change from the initial fast degradation rate to slower one was possibly caused by the aggregation as well as the surface passivation of nanoparticles [22,29].

### 3.4. Effect of the Source of Iron

**Figure 4:**
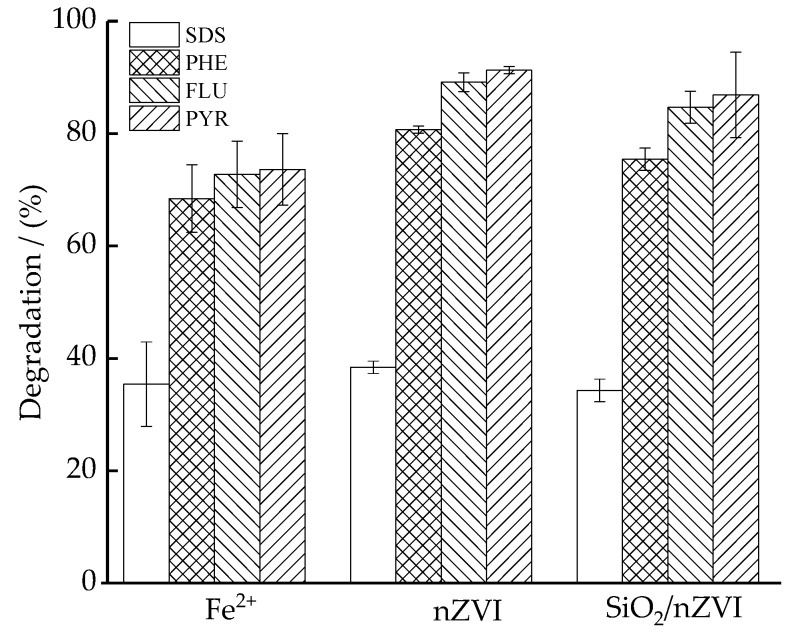
The degradation of PAHs and SDS in soil washing effluents with PS activated by different sources of iron (Fe^2+^, nZVI, and SiO_2_/nZVI). Values are presented as means and standard deviation.

As it was previously cited, iron was added as Fe^2+^, nZVI, or SiO_2_/nZVI. Figure 4 shows the degradation efficiencies of PHE, FLU, PYR, and SDS in soil washing effluents by activated PS. The degradation efficiencies of PAHs were higher for nZVI activation than Fe^2+^ activation, with a final degradation of PHE, FLU, and PYR of 84%, 89%, and 91%, respectively. This could be explained by the fact that nZVI produce a slower release of iron, minimizing the radical scavenger in Equation (5). Similar results were reported for the removal of PAHs and trichloroethylene with PS activated by Fe^2+^ [30,31]. In the case of SiO_2_/nZVI, the degradation efficiencies of PHE, FLU, and PYR were 75%, 85%, and 87%, respectively, which were not significantly different from those of nZVI system. There are contradictory results in the literature. According to Wan et al. [32], SiO_2_/nZVI showed an improvement of about 35% removal of 2,4-dichlorophenol compared to nZVI. However, no improvement in the PAH removal by SiO_2_/nZVI was observed in our study. The oxidation and agglomeration of nZVI can be well-suppressed by coating with SiO_2_, although the PAH removal efficiencies were not improved. Moreover, it is noted that the degradation efficiencies of FLU and PYR, with a ring number of 4, are higher than PHE, with a ring number of 3, which is consistent with the findings of Peluffo et al. [30]. It might be explained by the higher availability of PAHs with a higher ring number in freshly spiked soils and the reduced energy required to form the oxidation products [30,33]. It should be noted that there would be some difference in the degradation efficiency for soil effluents with different initial pollutant levels. Wei et al. [34] showed that the degradation efficiency increased with increasing initial contaminant concentration, but there was an optimal concentration at which the maximum removal efficiency could be obtained.

As for SDS, no significant difference was observed in the degradation efficiencies by PS activated with different sources of irons. The degradation efficiency of SDS was 34–38%, which is much lower than those of PAHs. The high residual of SDS makes it possible for the reuse of the treated effluent for subsequent washing processes. Similar results were reported in the treatment of soil washing effluents containing other organic pollutants and sulfates by activated PS. According to Tsitonaki et al. [35] and Long et al. [36], ${\mathrm{SO}}_{4}^{∙-}$ may oxidize benzene toluene ethylbenzene and xylene with a faster reaction rate than straight chain hydrocarbon surfactants that do not contain a benzene ring, so that SDS can be recycled from the soil washing water. In the mixture solution of nitrobenzene (NB) and sodium dodecyl benzene sulfonate (SDBS), the generated ${\mathrm{SO}}_{4}^{∙-}$ exhibited preferential degradation of NB by electron transfer over SDBS through hydrogen abstraction reactions [37].

### 3.5. Effect of the Dosage of SiO_2_/nZVI

**Figure 5:**
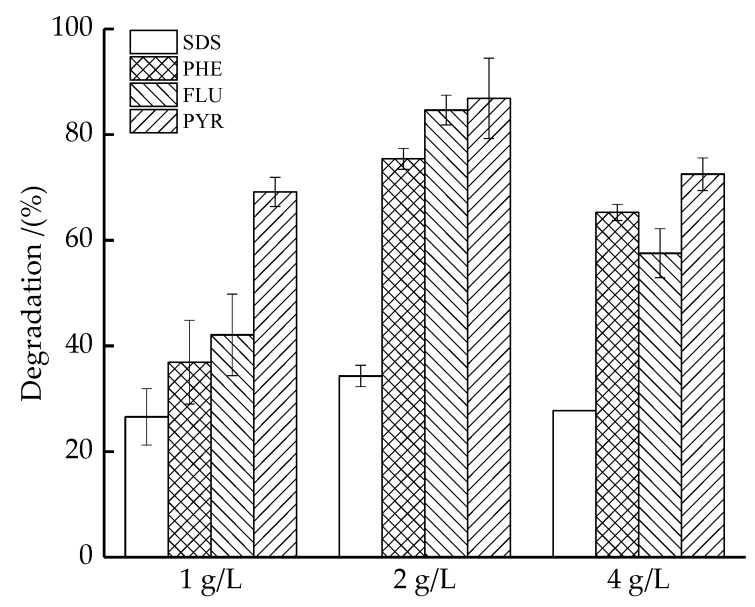
The degradation of PAHs and SDS in soil washing effluents by PS activated with different dosage of SiO_2_/nZVI (1, 2, and 4 g/L). Values are presented as means and standard deviation.

Figure 5 shows the degradation efficiencies of PAHs and SDS in soil washing effluents with PS activated by different dosages of SiO_2_/nZVI. For PHE, FLU, PYR, and SDS, the removal efficiencies were the highest at 2 g/L of SiO_2_/nZVI at equilibrium. It can be explained that more Fe^2+^ were released with increasing SiO_2_/nZVI, which facilitated the decomposition of PS to generate more ${\mathrm{SO}}_{4}^{∙-}$ (Equation (4)). Meanwhile, SiO_2_/nZVI can remove pollutants by itself. According to Wan et al. [32], SiO_2_ adsorption contributed 10% removal of 2,4-dichlorophenol. Nevertheless, the degradation of PAHs decreased when the dosage increased to 4 g/L. It can be inferred that the higher the dosage of SiO_2_/nZVI, the higher the PS consumption, which produced more sulfate radicals resulting in scavenging reactions (Equation (8)). In addition, PS and excess Fe^2+^ released from SiO_2_/nZVI (Equation (6)) could scavenge sulfate radicals (Equations (5) and (9)). The higher concentrations of SiO_2_/nZVI also made it easier to agglomerate with each other which may decrease the ability to activate PS.

(8) $${2\mathrm{SO}}_{4}^{∙-}\to S_{2}O_{8}^{2-}$$

(9) $${\mathrm{SO}}_{4}^{∙-}{+S}_{2}O_{8}^{2-}\to {3\mathrm{SO}}_{4}^{2-}$$

## 4. Limitations

In this study, PHE, FLU, and PYR were selected to represent PAHs with different hydrophobicity and molecular size. However, the three PAHs are not completely representative of the behavior of all PAHs as a whole due to the difference in physicochemical properties. There would be some difference in the washing efficiencies and cleanup of the breakdown products if higher molecular weight PAHs had been used, which needs to be studied further in the future.

Aging time has a major effect on the removal of PAHs by soil washing. Longer aging times are likely to be more representative of real-world contaminated soils. However, freshly spiked soil was used in this paper which mainly focused on the treatments of soil washing effluents. 

In this study, nZVI synthesized in a single batch was used for the degradation experiments. However, batch to batch reproducibility of experimental results can be quite poor for nanomaterials. Batch-to-batch reproducibility of the results was not tested, which is also a limitation of the study.

In spite of the above limitations of the study, this paper provides important information for the treatment of soil washing effluents containing SDS and PAHs.

## 5. Conclusions

PAHs can be effectively removed from the soil by soil washing using SDS as the surfactant. The removal efficiencies of PAHs were 37–44% at an SDS concentration of 20 g/L. PS activated by different sources of iron (Fe^2+^, nZVI, and SiO_2_/nZVI) can effectively degrade PAHs in soil washing effluents. Among the three sources of iron, SiO_2_/nZVI had a comparable ability to nZVI in the degradation of PAHs and SDS. The oxidation and agglomeration of nZVI can be well-suppressed by coating with SiO_2_, which makes it more preferable in practice. At the dosage of 2 g/L (in the amount of iron) of SiO_2_/nZVI, the removal efficiencies of PHE, FLU, PYR, and SDS within 30 min of treatment were 75%, 85%, 87%, and 34%, respectively. Moreover, the degradation of SDS was much lower than those of PAHs in soil washing effluents, which facilitated the recycling of SDS. It is suggested that the combination of soil washing with SDS and iron-activated persulfate oxidation is a feasible route in the remediation of PAH-contaminated soil.

## Figures and Tables

**Table 1:** Main properties of the soil.

Property	Value
Sand (%)	53
Silt (%)	24
Clay (%)	22
pH	6.8
Organic carbon (%)	0.8
Cation exchange capacity (cmol·kg^−1^)	6.8
